# Identification of Exoenzymes Secreted by Entomopathogenic Fungus *Beauveria pseudobassiana* RGM 2184 and Their Effect on the Degradation of Cocoons and Pupae of Quarantine Pest *Lobesia botrana*

**DOI:** 10.3390/jof8101083

**Published:** 2022-10-14

**Authors:** Matias Arias-Aravena, Fabiola Altimira, Daniela Gutiérrez, Jian Ling, Eduardo Tapia

**Affiliations:** 1 Laboratorio de Entomología y Biotecnología, Instituto de Investigaciones Agropecuarias, INIA La Platina, Santiago 8831314, Chile; 2 Facultad de Medicina Veterinaria y Agronomía, Universidad de Las Américas, Santiago 8370040, Chile; 3 Department of Plant Protection, Institute of Vegetables and Flowers, Chinese Academy of Agricultural Sciences, Beijing 100081, China

**Keywords:** entomopathogenic fungi (EPF), *Beauveria pseudobassiana*, exoenzymes, cocoon, *Lobesia botrana*, cold-adapted protein

## Abstract

*Beauveria pseudobassiana* RGM 2184 has shown 80% maximum efficacy against the pest *Lobesia botrana* in the autumn and winter seasons. This suggests that the strain possesses an interesting battery of enzymes that are cold-adapted to penetrate the thick and hydrophobic cocoon of *L. botrana*. In this study, screening of the proteolytic, lipolytic, and chitinolytic activity of enzyme extracts secreted by the RGM 2184 strain was carried out in various culture media. The enzyme extracts with the highest activity were subjected to zymography and mass spectrometry. These analyses allowed the identification of two proteases, two lipases, and three chitinases. Comparative analysis indicated that the degree of similarity between these enzymes was substantially reduced when the highest degree of taxonomic relatedness between RGM 2184 and the entomopathogenic fungus strain was at the family level. These results suggest that there is a wide variety of exoenzymes in entomopathogenic fungi species belonging to the order Hypocreales. On the other hand, exoenzyme extract exposure of cocoons and pupae of *L. botrana* provoked damage at 10 °C. Additionally, an analysis of the amino acid composition of the RGM 2184 exoenzyme grouped them close to the cold-adapted protein cluster. These results support the use of this strain to control pests in autumn and winter. Additionally, these antecedents can form a scaffold for the future characterization of these exoenzymes along with the optimization of the strain’s biocontrol ability by overexpressing them.

## 1. Introduction

Entomopathogenic fungi (EPF) are parasitic microorganisms with the ability to infect and kill arthropods. They are mainly used as biopesticides in crops as a safe and efficient alternative to toxic chemical insecticides [1,2]. There are more than 750 species of EPF, belonging to 85 genera and infecting 20 of the 30 insect orders described to date [3,4]. The genera *Metarhizium*, *Beauveria*, *Cordyceps*, and *Akanthomyces*, which belong to the order Hypocreales, are the most commonly used for pest control because they are relatively easy to grow en masse, have a wide range of hosts, and exhibit similar efficacy to commercial insecticides [5,6,7].

Unlike viruses and bacteria, EPF infect insects by directly penetrating the cuticle, so it is not necessary for insects to ingest the spores. The mechanism of infection of EPF is described by five main stages: (1) conidia (spore) adhesion, (2) conidia germination, (3) cuticle penetration, (4) hemolymph colonization, and (5) emergence and sporulation. The first stage occurs when the conidia are deposited on the cuticle of the arthropod and adhere through hydrophobic interactions [3]. Conidia germination occurs in the carbon and energy sources present in the insect cuticle under temperature and humidity conditions that are strain-specific [8]. The third stage begins with the formation of the appressorium. This structure generates the mechanical rupture of the arthropod cuticle, but it is insufficient for the EPF to penetrate such a complex structure [9]. The pathogen must be able to secrete lytic enzymes capable of degrading the different polymers that form the cuticle. The pathogenicity of the insect depends mainly on this enzyme pool and its efficiency [10]. Once inside the hemolymph, the entomopathogenic fungus colonizes and multiplies in a yeast-like manner. When the nutrients are depleted, the fungus finally emerges and acquires its sporulating capacity [11].

As noted, lytic enzymes play the most important role during the process of insect infection by EPF. Given the cuticle components, the fungus must release lipases, proteases, and chitinases. The first to act are lipases due to the components of the epicuticle (outer cuticle) [12]. A heterogeneous mixture of lipids, long-chain alkenes, esters, and fatty acids is the main constituent of this layer [12]. Its lytic activity increases the adhesion of germ spores to insect cuticles by increasing hydrophobic interactions between the fungus and the cuticle surface [12]. The proteases subsequently cleave the peptide bonds of the sclerostin proteins present in the exocuticle. The most described are subtisilin-like proteases called Pr1 (serine endoproteases). They are considered a virulence factor because in addition to degrading cuticle proteins, they also modify the cuticle surface and facilitate adhesion [3]. Due to the action of enzymes with proteolytic activity, chitin fibers are exposed. Therefore, the last ones to act temporarily are the chitinases [12]. For efficient rupture of the fibers, exochitins and endochitinases must act together. The study of the enzymatic tools entomopathogenic fungi use to carry out their insecticidal action has allowed the optimization of methods to determine increased EPF virulence by the overexpression or fusion of enzymes with different lytic activity [9]. Identifying and characterizing the enzymes of different EPF could allow mixtures of these microorganisms with their exoenzymes to achieve more efficient formulations.

*Lobesia botrana* (Denis and Schiffermüller) (Lepidoptera: Tortricidae) is a quarantine pest affecting the export supply of table grapes. The larvae of this species feed on fruit berries. The perforations allow the release of sugary juices from inside the fruit, creating conditions for the growth of opportunistic phytopathogenic fungi (i.e., *Botritys cinerea*). Increasing production costs, due to fungicide investment and economic losses due to impoverishment of fruit quality. In the autumn–winter season, the pupae are in diapause, presenting a thick and highly hydrophobic cocoon. This tissue protects the pupae from low temperatures, water, predators, and even agrochemicals. Formulations using the entomopathogenic fungus *Beauveria pseudobassiana* S.A Rehner and Humber (Hypocreales: Cordycipitaceae) as the active ingredient have demonstrated the ability to control *L. botrana* in the diapause stage and to penetrate its cocoon’s silk (Figure S1) [13]. A wettable powder formulation of the *B. pseudobassiana* RGM 2184 strain was shown to achieve a maximum efficacy level of 80% against pupae of *L. botrana* in field trials performed in two regions of Chile over autumn and winter [7]. In order to obtain an approximation of the enzymatic tools used by strain RGM 2184 to penetrate the cocoons and pupae of *L. botrana* at low temperatures, the objectives in this study were as follows: (1) to identify possible genes and/or gene clusters in the genome of *B. pseudobassiana* RGM 2184 encoding enzymes involved in its biocontrol activity; (2) to identify the enzymes secreted by the fungus RGM 2184; (3) to evaluate homologues of these genes in other EPF through comparative genomic analysis; and (4) to evaluate the insecticidal activity of exoenzymes secreted by strain RGM 2184 at low temperatures.

## 2. Materials and Methods

### 2.1. Biological Materials and Culture Media

Pupae and cocoons of *L. botrana* were obtained from the *Lobesia botrana* National Program (PN*Lb*; in Spanish, Programa Nacional de *Lobesia botrana*, Santiago, Chile) of the Agriculture and Livestock Service (Servicio Agrícola y Ganadero, Santiago, Chile). EPF strain *B. pseudobassiana* RGM 2184 was obtained from the Chilean Culture Collection of Microbial Genetic Resources of the Agricultural Research Institute (Instituto de Investigaciones Agropecuarias, Santiago, Chile). Strain RGM 2184, isolated from southern Chile, is native to areas with low temperatures and high humidity [13].

The culture media used in this study were as follows: M1 (4.9 g/L dextrose, 2.30 g/L yeast extract); M2 (M1 supplemented with mineral salts [7]); PDB (potato dextrose broth; BD, USA); G55 (glucose-based medium, 0.3 g/L dextrose, 0.1 g/L bacteriological peptone, 0.02 g/L tryptone, 0.02 g/L yeast extract, 0.06 g/L sodium chloride, 0.005 g/L magnesium chloride, and 0.007 g/L L-methionine [14] adjusted to pH 5.5); G8 (glucose-based medium, 0.3 g/L dextrose, 0.1 g/L bacteriological peptone, 0.02 g/L tryptone, 0.02 g/L yeast extract, 0.06 g/L sodium chloride, 0.005 g/l magnesium chloride, and 0.007 g/L L-methionine adjusted to pH 8.0)); A55 (starch-based medium,10 g/L starch, 10 g/L yeast extract [14] adjusted to pH 5.5); A8 (starch-based medium,10 g/L starch, 10 g/L yeast extract adjusted to pH 8.0); CN55 (intermediate C/N ratio, 40 g/L dextrose, 10 g/L bacteriological tryptone [15] adjusted to pH 5.5) and CN8 (intermediate C/N ratio, 40 g/L dextrose, 10 g/L bacteriological tryptone adjusted to pH 8); and YSM (20 g/L sucrose, 5 g/L yeast extract, 1.5 g/L KH_2_PO_4_, 0.5 g/L MgSO_4_∙7H_2_O, 0.01 g/L CaCl_2_, 0.00003 g/L H_3_BO_3_, 0.00004 g/L MnSO_4_∙4H_2_O, 0.000025 g/L Na_2_MoO_4_∙2H_2_O, 0.00008 g/L CuSO_4_∙5H2O, 0.0004 g/L ZnSO_4_∙7H_2_O, 0.0005 g/L FeCl_3_∙6H_2_O, 0.0004 g/L CoCl_2_∙6H_2_O [16]). All media were supplemented with 1.2% Tween 80 (Sigma-Aldrich, St. Louis, MO, USA.).

### 2.2. Extraction of Exoenzymes

Conidia from PDA cultures at 7 d were suspended in sterile 0.9% NaCl supplemented with 0.05% Tween 80. For 10 different media (M1, M2, PDB, G55, G8, A55, A8, CN55, CN8, and YSM), a suspension of 10^6^ conidia/mL was used in 80 mL of each media. The cultures were incubated at 200 rpm and 25 ± 2 °C for 7 d and then centrifuged at 5000× *g* for 10 min at 20 °C. Propagule supernatants were collected, filtered using a 0.45 µm filter unit (Millipore, Burlington, MA, USA), and incubated with 3 volumes of cold acetone for 1 h at −20 °C. The supernatant from each tube was removed after centrifugation at 13,000× *g* for 15 min at 4 °C, and the corresponding pellets were resuspended in 1 mL of 25 mM Tris–HCl (pH 7.5) and 150 mM NaCl. Proteins were determined using a Qubit Protein Assay Kit (Thermo Fisher, Waltham, MA, USA).

### 2.3. Preliminary Evaluation of the Presence of Exoenzymes in Protein Extracts

To determine the presence of exoproteases, exolipases, and exochitinases in the protein extracts obtained from the supernatant of *Beauveria pseudobassiana* RGM 2184 growth in the different culture media (Section 2.1), semiquantitative colorimetric enzymatic assays were performed. Proteolytic, lipolytic, and chitinolytic activity was evaluated according to the assays described by Secades and Guijarro [17], Izrael-Zivkovic et al. [18], Kim et al. [19], respectively. Absorbance values of enzymatic assays were recorded and compared. The supernatant cultures with the highest absorbance values were selected. The M2 medium was selected for the identification of proteases and lipases. The A8 medium was selected for the identification of chitinases and lipases.

### 2.4. Detection of Exoproteases, Exolipases, and Exochitinases

*Electrophoretic assay:* Electrophoresis was performed under denaturing conditions (SDS-PAGE). The protein extract obtained from the fungal culture supernatant (M2 or A8) was mixed with 5× loading buffer (60 mM Tris/HCl, 25% *v/v* glycerol, 2% *w/v* SDS, and 0.1% *w/v* bromophenol blue, pH 6.8). Then, 20 µL of the resulting solution (35 µg) was separated using 12% (*w*/*v*) denaturing polyacrylamide gel electrophoresis, as described by Laemmli (1970) [20].

*Zymography for proteases:* Proteases present in the protein extract of the supernatant of M2 medium were detected by electrotransfer zymography performed in a semi-wet electrotransfer chamber (Trans-Blot^®^ SD Semi-Dry Transfer Cell). To renaturate the proteins, they were incubated in a 50 mM Tris–HCL buffer solution, pH 7.4, with 2.5% Triton X100 for 1 h. Subsequently, the gel was confronted with a gel copolymerized with 0.2% gelatin. Electrotransfer was performed at 15 V for 20 min. To allow protease activity, the gel was incubated in the presence of a buffer with co-factors (6.055 g/L Tris-HCl, 11.69 g/L NaCl, 0.07 g/L ZnCl_2_, 0.74 g/L CaCl_2_·2H_2_O, and 0.2 g/L NaN_3_, pH 7.4) for 12 h at 25 °C. For detection, the gel was stained with Coomassie blue for 1 h. Proteolytic activity was assessed by visualization of a transparent area on the blue background of the stained gel [21,22].

*Zymography for lipases:* For the detection of lipases in the protein extract of RGM 2184 culture supernatant in M2 medium, capillary transfer zymography was performed. To renaturate the enzymes, the gel was incubated in 50 mM Tris–HCL buffer, pH 7.4, with 2.5% Triton X100 for one hour. The gel was then confronted with a gel containing 12% acrylamide and 2% tributyrin. The gels were placed on top of absorbent paper towels (top gel co-polymerized with tributyrin), in contact with a buffer solution with co-factors (6.055 g/L Tris–HCl, 11.69 g/L NaCl, 0.74 g/L CaCl_2_·2H_2_O, and 0.2 g/L NaN_3_, pH 7.4) to induce lipase activity [22].

### 2.5. Mass Spectrometry Analysis of Protein Extracts A8 and M2

Protease/phosphatase inhibitor at 1× concentration was added to 500 µg of protein from supernatant extract of RGM 2184 in A8 and M2. The samples were subsequently lyophilized and resuspended in 8 M urea and 25 mM ammonium bicarbonate at pH 8. The extract solutions were then quantified by Qubit 4TM using the Qubit Protein Assay Kit (Thermo Scientific, Waltham, MA, USA). Once quantified, they were precipitated with cold acetone and allowed to sit overnight at −80 °C. They were centrifuged at 16,000× *g* for 15 min at 4 °C, and the supernatant was discarded. The samples were resuspended in 8 M urea and 25 mM ammonium bicarbonate. They were then reduced with DTT and subsequently alkylated by adding iodoacetamide to a final concentration of 20 mM in 25 mM ammonium bicarbonate and incubated for 1 h in the dark at room temperature. Then, 100 µg of protein was taken for the digestion process. The digestion was performed with sequencing grade Trypsin (#V5071; Promega, Madison, WI, USA) in a 1:50 ratio with protease and incubated for 16 h at 37 °C, and the digestion reaction was stopped by pH by adding 10% formic acid. The samples then underwent clean-up using solid-phase extraction with a Sep-Pak Vac C−18 column (Waters™, Milford, MA, USA), following the manufacturer’s instructions. The resulting peptides were resuspended in ultrapure water with 0.1% formic acid of LC-MS/MS quality and quantified using the Direct Detect system (Merck Millipore, Burlington, MA, USA). Then, 200 ng of the peptides obtained in the previous step were injected into a nanoUHPLC nanoElute (Bruker Daltonics, GmbH, Bremen, Germany) coupled to a timsTOF Pro (trapped ion mobility spectrometry–quadrupole time-of-flight) mass spectrometer (Bruker Daltonics) using an Aurora series UHPLC column from IonOpticks (75 um × 100 mm, C18, 1.9 A). The results were collected using timsControl 2.0 software (Bruker Daltonics) with 10 PASEF cycles and analyzed using PEAKS Studio X+ software (Bioinformatics Solutions, Columbia, Waterloo, CA, USA). The database used for identification was proteins predicted from the *Beauveria pseudobassiana* RGM 2184 genome (JAKJXD000000000.1) [7]. This mass spectrometry analysis allowed the identification of lipases, proteases, and chitinases secreted by strain RGM 2184 in the A8 and M2 culture media. The nucleotide sequences of detected enzymes have been deposited in Genbank under accession numbers OP328424, OP328429, OP328425, OP328426, OP328430, OP328427, and OP328428, for P1, P2, Lip1, Lip2, Chi1, Chi2, and Chi3, respectively.

### 2.6. Comparative Analysis of Exoenzyme Sequences

The sequences of exoproteases, exolipases, and exochitinases of *B. pseudobassiana* RGM 2184 were aligned with characterized and/or predicted exoenzymes of other EPF strains using the MUSCLE method [23]. The relatedness of exoenzymes was assessed by phylogenetic analysis carried out using the neighbor-joining method [24] implemented in MEGA7 software [25]. Evolutionary distances were computed using the p-distance method, with the number of amino acid differences per site used as the unit [26]. Support for the hypothesis of relationships was assessed using 1000 bootstrap replicates [27].

The Blast score ratio (BSR) test was conducted to evaluate homologies between extra-cellular enzyme (exoenzyme) sequences of *B. pseudobassiana* RGM 2184 identified by MS (Section 2.4) regarding predicted proteins from 27 entomopathogenic fungi sequenced (Table S1). The BSR is calculated by dividing the query bit score by the reference bit score, resulting in a value between 0.0 and 1.0 [28]. A score of 1 indicates a perfect match and a score of 0 indicates no Blast match of a queried protein (exoenzymes of RGM 2184). Values over 0.4 indicate the presence of a protein homologue [28]. The normalized pairs of BSR indices were plotted using R software (v. 4.0.1).

### 2.7. Comparative Analysis of Amino Acid Composition

The amino acid composition of thermophilic, mesophilic, and cold-adapted proteins described in the literature was determined using the Composition-Based Protein Identification (COPid) server (https://webs.iiitd.edu.in/raghava/COPid/whole_comp.html, accessed on: 2 August 2022) (Table S2). For each enzyme group (thermophilic, mesophilic, or cold-adapted), the average percentage and standard deviation were calculated. Subsequently, a t-test was performed to determine statistically significant differences between the three groups of enzymes for each amino acid (Table S3), followed by the two-stage linear step-up procedure of Benjamini, Krieger, and Yekutieli, with Q = 1% [29]. The amino acids that showed differences were used as the axis for the construction of a 3D scatter plot using R version 4.2.1 with the car and rgl packages. The average percentages of amino acids in the exoenzymes of strain RGM 2184 were incorporated in this analysis.

### 2.8. Evaluation of Exoenzymes on L. botrana Cocoon and Pupae

For this evaluation, 138 µg of exoenzyme extract from the supernatants of RGM 2184 cultures in A8 and M2 media and a mixture of both extracts (60 µL total volume) were added over *L. botrana* pupae and cocoon silk squares (approximately 5 × 5 mm) in 1.5 mL microcentrifuge tubes. The control treatment consisted of exposing *L. botrana* pupae and cocoon silk to 60 µL of resuspension buffer (25 mM Tris with 150 mM NaCl, pH 7.0) without RGM 2184 exoenzymes. All treatments were performed in triplicate and were incubated at 10 ± 2 and 25 ± 2 °C for 7 days. After incubation, the samples were fixed with 3% glutaraldehyde in 0.268 M sodium cacodylate buffer (pH = 7.0), followed by dehydration, critical point drying, and gold-coating on a 0.22 mm polycarbonate membrane. The samples were visualized using a TM 3000 scanning electronic microscope (SEM) (Hitachi, Tokyo, Japan).

## 3. Result and Discussion

During autumn and winter, individuals belonging to the order Lepidoptera are in the pupal stage, wrapped in a dense cocoon that prevents penetration by natural enemies and agrochemicals [7]. The cocoon is mostly composed of a water-insoluble crystalline fibrous protein called fibroin, with a smaller proportion of an amorphous matrix of a water-soluble globular protein called sericin [30]. The cocoon allows the pupa within to evade predators and agrochemicals, because they cannot adhere to or penetrate the cocoon. The cuticle of the pupa is mainly composed of hydrocarbons, lipids (free fatty acids and wax esters), chitin fibers, and proteins [31]. Both the cocoon and the pupa are composed of fibers susceptible to degradation by hydrolytic enzymes secreted by entomopathogenic fungi.

**Figure 1:**
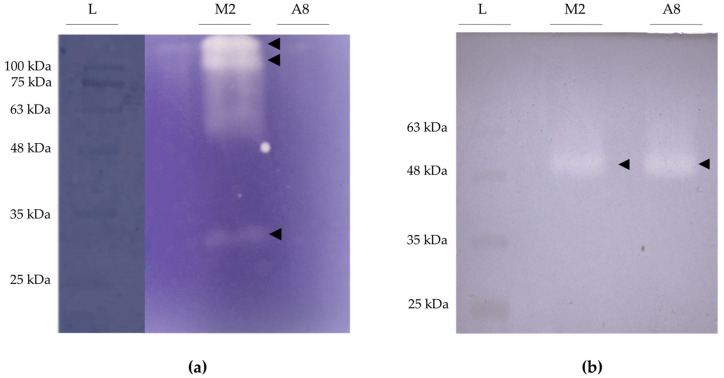
Detection by zymography of proteases and lipases secreted by *B. peudobassiana* RGM 2184. (**a**) Gel copolymerized with gelatin for the detection of proteases; whitish bands indicate the presence of a protease. (**b**) Gel copolymerized with tributyrin for the detection of lipases; bright bands indicate the presence of lipases. L: ladder lane; M2: enzyme-enriched supernatant extract from RGM 2184 culture in M2 medium; A8: enzyme-enriched supernatant extract from RGM 2184 culture in A8 medium.

**Figure 2:**
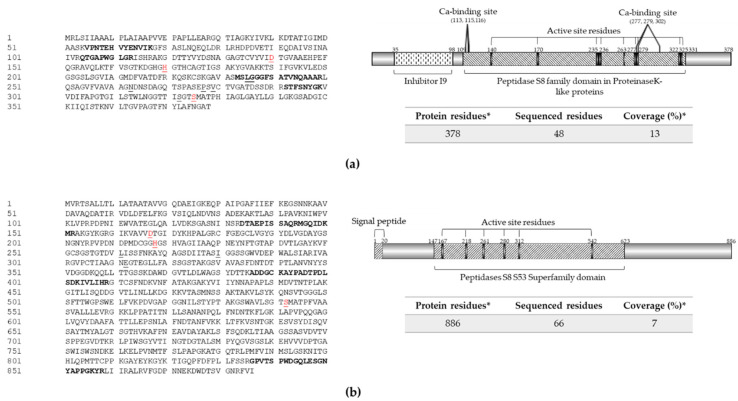
Extracellular proteases identified in protein extract from strain RGM 2184 culture supernatant in M2 medium. (**a**,**b**) Sequences and scheme of exoproteases P1 (OP328424) and P2 (OP328429), respectively. Exoprotease sequences (left panel) were predicted from the *B. pseudobassiana* RGM 2184 genome (JAKJXD000000000.1). Peptides sequenced by mass spectrometry are highlighted in bold in the left panel. Relevant domains for these proteins (right panels) were detected though the NCBI conserved domains database. Signal peptide (italic), active site residues (underlined), and catalytic triad (red) are indicated in the left and right panels, respectively. * indicates predicted information.

The strain *B. pseudobassiana* RGM 2184 was able to control *L. botrana* in the stage when the pupa was coated by a thick cocoon at low temperature (0–15 °C) in two autumn–winter seasons in Chile [13]. Unlike agrochemicals, this EPF species can penetrate the cocoon and cuticle of *L. botrana* pupae (Figure S1). Additionally, this strain was found to encode a battery of extracellular enzymes that can act as pathogenicity factors on arthropods by degrading these tissues [7]. For this reason, screening of proteolytic, lipolytic, and chitinolytic activity of protein extracts from supernatants of cultures of strain RGM 2184 grown in media at different pH was carried out (Figure S2). The highest proteolytic and lipolytic activity was detected in the culture on M2 medium (based on glucose, yeast extract, and salts, pH = 5.5), while the highest chitinolytic activity was recorded on A8 medium (starch-based, pH = 8.0) (Figure S2). Three proteases (~36, ~105, and ~113 kDa) were detected by zymography assay from the supernatant extract of RGM 2184 in M2 medium (Figure 1a). Two of them (P1 and P2) were identified by mass spectrometry (Figure 2). These proteases belong to a subtilisin-like Pr1 protease complex described as a cuticle-degrading enzyme and activator of the insect prophenoloxidase cascade [29]. Phylogenetic analysis of P1 and P2 protein sequences indicated that they are closely related to Pr1B2 and Pr1C, respectively, described in *B. bassiana* ARSF 2860 (Figure S3). The third protease detected by zymography corresponded to an isoform of Pr1C. Isoforms of this enzyme have been described in *Metarhizium* strains [32,33].

An analysis of the amino acid sequence of these enzymes allowed us to find potential motifs and domains that could be involved in the functionality, stability, and mechanism of secretion into the extracellular space (Figure 2). P1 contains the S8 peptidase domain proteinase K-like, which presents an Asp/His/Ser catalytic triad that is characteristic of subtilisin-type serine proteases (Figure 2a). In addition, the enzyme sequence has a Ca-binding site. The presence of bound Ca ions is a feature shared by members of the subtilisin superfamily, in which calcium binding has been shown to be essential for correct folding and structural stability [34,35,36,37].

There is significant evidence that a homologue of Pr1B2 is one of the pathogenetic factors secreted by *Metarhizium anisopliae* and *Beauveria bassiana*. Indeed, the encoding gene of Pr1B2 in *B. bassiana* ARSEF 2860 (BBA_00443) was one of the 100 most highly expressed genes of this strain in insect cuticle and hemolymph from locust (*Locusta migratoria*) [37]. This antecedent indicates the importance of this enzyme’s role as a pathogenicity factor. On the other hand, P2 detected in the supernatant extract showed high identity and conserved domains present in members Pr1C of subtilisin-like Pr1 proteases.

Additionally, studies carried out by [32] showed that Pr1B2 and PR1C, along with three other conserved members of the Pr1 protein (Pr1G, Pr1A2, and Pr1B1), are essential for the maintenance of total extracellular PR1 activity required for cuticle degradation during hyphal invasion of *B. bassiana* into host insect. Subtilisins are particularly attractive because they display broad substrate specificity and high stability at neutral and alkaline pH [38,39].

**Figure 3:**
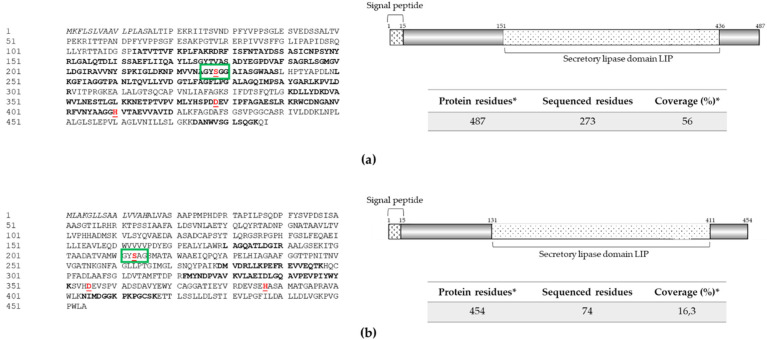
Extracellular lipases identified in protein extract from strain RGM 2184 culture supernatant in M2 and A8 media. (**a**,**b**) Sequences and scheme of exolipases Lip1 (OP328425) and Lip2 (OP328426), respectively. Exolipase sequences (left panel) were predicted from *B. pseudobassiana* RGM 2184 genome (JAKJXD000000000.1). Peptides sequenced by mass spectrometry are highlighted in bold (left panel). Relevant domains for these proteins (right panels) were detected though the NCBI conserved domains database. Signal peptide (italic), active site residues (underlined), catalytic triad (red), and relevant motive (green rectangles) are indicated in left and right panels, respectively. * indicate predicted information.

On the other hand, the lipolytic activity zymograms of protein-enriched supernatant fraction from the RGM 2184 culture in M2 and A8 exhibited a band of approximately 57 kDa (Figure 1b). Concomitantly, mass spectrometric analysis indicated that these extracts possessed two lipases of similar molecular weight but with different sequences (Figure 3). Therefore, it is likely that both lipases were not visualized by zymography because they overlapped due to having a similar molecular weight.

Those enzymes were named Lip1 and Lip2, and their sequences were compared in phylogenetic analysis with other lipases described and/or predicted in EPF strains. Lip1 shares the same clade (Figure S3b) as Bbl1 lipase described in *B. bassiana* bbl1 [40]. Lip2 was grouped in the same clade as other EPF lipases that have been predicted but not yet characterized (Figure S3b).

The sequence of Lip1 and Lip2 showed a pfam03583 domain that is found in bacterial lipases expressed and secreted during the infection cycle of these pathogens (NCBI Conserved Domain page; [41]) (Figure 3). Both enzymes exhibited the GXSXG motif (where X can be any amino acid) and the catalytic triad Ser, Asn, and His in their sequences: Ser^226^, Asn^372^, and His^404^ in P1 and Ser^214^, Asn^399^, and His^431^ in P2 (Figure 3). The lipases secreted by strain RGM 2184 participate in the hydrolysis of insect integument components (e.g., ester bonds of lipoproteins, fats, and waxes) and/or degrade the insect haemocoel, which has a lipid composition of 1.5%–5.5% (*w*/*v*) [12].

**Figure 4:**
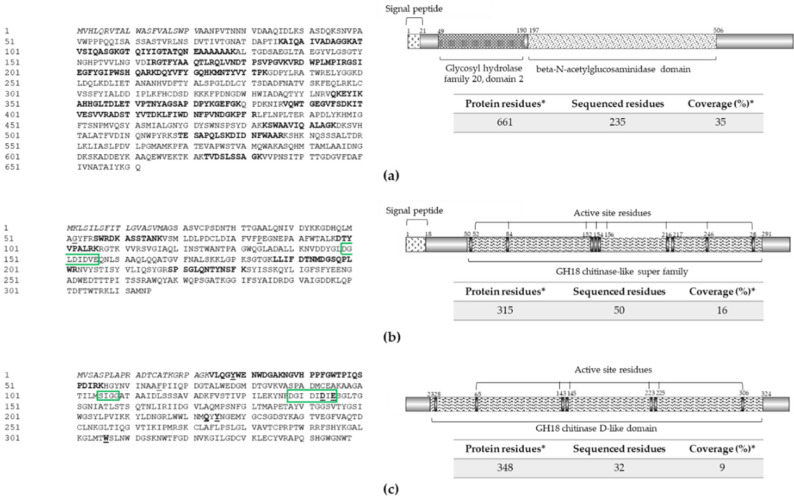
Extracellular chitinases identified in protein extract from strain RGM 2184 culture supernatant in A8 and/or M2 media. (**a**–**c**) Sequences and scheme of exochitinases Chi1 (OP328430), Chi2 (OP328427), and Chi3 (OP328428), respectively. Exochitinase sequences (left panel) were predicted from the *B. pseudobassiana* RGM 2184 genome (JAKJXD000000000.1). Peptides sequenced by mass spectrometry are highlighted in bold (left panel). Relevant domains for these proteins (right panels) were detected though the NCBI conserved domains database. Signal peptide (italic), active site residues (underlined), and relevant motives (green rectangle) are represented in left and right panels, respectively. * indicate predicted information.

Additionally, the highest chitinolytic activity was detected in protein-enriched supernatant fraction of strain RGM 2184 grown in A8 medium (Figure S2c). In this extract, the enzymes identified by mass spectroscopy were named Chi1 (~71 kDa), Chi2 (~34 kDa), and Chi3 (~37 kDa) (Figure 4). On the other hand, in M2 extract Ch1 and Chi2 exoenzymes were identified. However, zymography could not be performed to detect them due to assembly problems with this technique.

Chi2 showed the glycosyl hydrolase family 18 (GH18) catalytic domain, characteristic of endo-beta-N-acetylglucosaminidases (Figure 4b). This type of enzyme has been characterized mainly in bacteria (NCBI; [41]) and predicted in *B. bassiana* ARSEF 2860 (XP008597133.1) and *Cordyceps militaris* CM01 (XP006673222.1). The sequences of Chi3 showed high identity with Bbchit1, described in *B. bassiana*. Both enzymes belong to the chitinase family 18, and have similar substrate binding and catalytic domains (SXGG and DXXDXDXE) (Figure 4b,c). Bbchit1 was previously characterized. It is an endochitinase that has a molecular mass of about 33 kDa and pI of 5.4 [39]. Overproduction of Bbchit1 enhanced the virulence of *B. bassiana* against aphids, as indicated by significantly lower 50% lethal concentration and 50% lethal time of transformants compared to the values for the wild-type strain [42].

**Figure 5:**
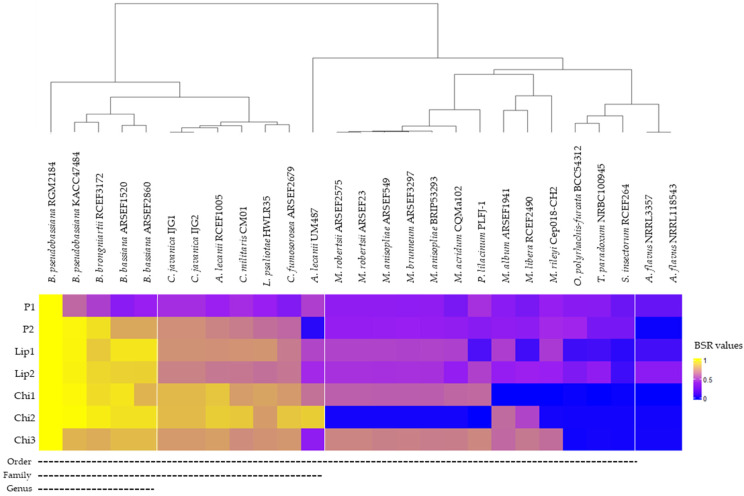
Heat map displaying similarity degree between identified exoenzyme of *B. pseudobassiana* RGM 2184 in comparison to other entomopathogenic fungi. A score of 1 indicates a perfect match and a score of 0 indicates no BLAST match for a query enzyme in the reference genome. Values greater than 0.4 indicate the presence of a homologous enzyme. Segmented lines indicate that EPF strains share the same taxonomic level.

Comparative genomic analysis of the exoenzymes of strain *B. pseudobassiana* RGM 2184 between sequenced EPF strains indicated that there is a significant proportion of homologous genes encoding these exoenzymes in strains that belong to the same genus and species as RGM 2184 (Figure 5). However, the number of homologous exoenzymes was significantly reduced when the highest degree of taxonomic relatedness between RGM 2184 and EPF strains was at the family or order level (Figure 5). These results suggest that there is a wide variety of lipases, proteases, and chitinases in the family’s taxonomy belonging to the order Hypocreales, such as Cordycipitaceae (e.g., *Beauveria* spp.) and Clavicipitaceae (e.g., *Metarhizium* spp.). Similar findings of phylogenetic analysis of chitinase Bbchit1 sequences from insect-pathogenic fungal strains belonging to *B. bassiana* and *Metarhizium* spp. Have been reported [42]. A large phylogenetic distance between chitinase sequences of these strains was observed. This background is relevant to biocontrol strategies, because a mixture of strains belonging to *Beauveria* and *Metarhizium* genera could enhance the enzymatic repertoire exerting action on pests. This could contribute to control the pest efficiently.

**Figure 6:**
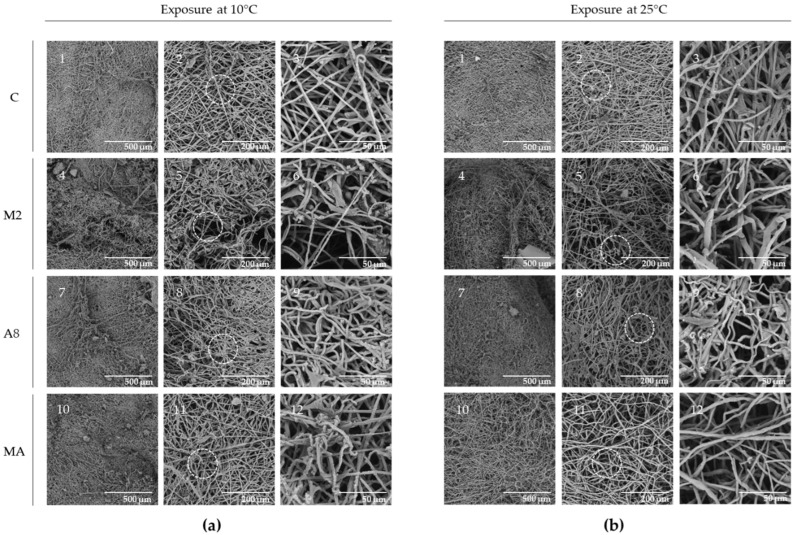
SEM micrographs of *L. botrana* cocoon exposed to RGM 2184 protein extract supernatants: (**a**) treatments incubated at 10 °C; (**b**) treatments incubated at 25 °C. Row C (1, 2, 3) shows the control treatment; row M2 (4, 5, 6) shows cocoons exposed to enzyme extract obtained from strain RGM 2184 supernatant in M2 culture medium; row A8 (7, 8, 9) shows cocoons exposed to enzyme extract obtained from strain RGM 2184 supernatant in A8 culture medium. Row MA (10, 11, 12) shows cocoons exposed to a mixture of enzyme extracts from strain RGM 2184 supernatant grown in M2 and A8 culture media (1:1). Circles indicate zoomed-in areas (images in the third column of each panel).

**Figure 7:**
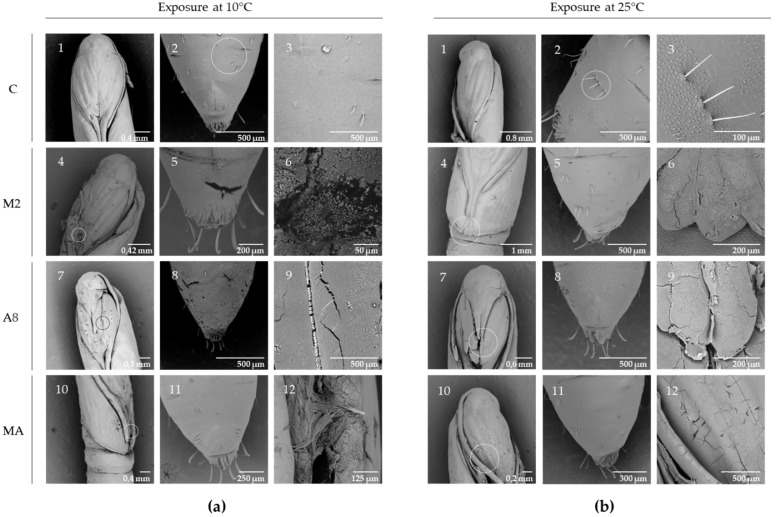
SEM micrographs of individual *L. botrana* pupae exposed to RGM 2184 protein extract cell supernatant: (**a**) incubated at 10 °C, (**b**) incubated at 25 °C. Row C (1, 2, 3) shows the control treatment; row M2 (4, 5, 6) shows pupae incubated with enzyme extract obtained from RGM 2184 supernatant in M2 culture medium; row A8 (7, 8, 9) shows pupae incubated with enzyme extract obtained from RGM 2184 supernatant in A8 culture medium; row MA (10, 11, 12) shows pupae incubated in a mixture of enzyme extracts from RGM 2184 supernatant grown in M2 and A8 culture media (images in the third column of each panel).

The effect of RGM 2184 exoenzymes on the degradation of cocoon and cuticle of *L. botrana* pupae in autumn–winter temperature was visualized by SEM analysis. Cocoon tissue exposed to enzyme extract of supernatant culture from M2 containing protease showed markedly less tissue compaction and density compared to other treatments at low temperature (10 °C) and room temperature (25 °C) (Figure 6). On the other hand, pupae exposed to enzyme extract of supernatant culture from M2, A8, and a mixture of both showed surface degradation, perforations, and cuticular rupture, while the control treatments did not show such damage at the same temperatures (Figure 7). Therefore, the enzymatic degradation of cocoon and cuticle tissue of *L. botrana* by the enzymes secreted by strain RGM 2184 at 10 °C was consistent with the strain’s ability to colonize *L. botrana* in the fall–winter season [13]. This result and the ability to grow at 4 °C (data not shown) suggest that strain RGM 2184 secretes enzymes that are cold-adapted.

**Figure 8:**
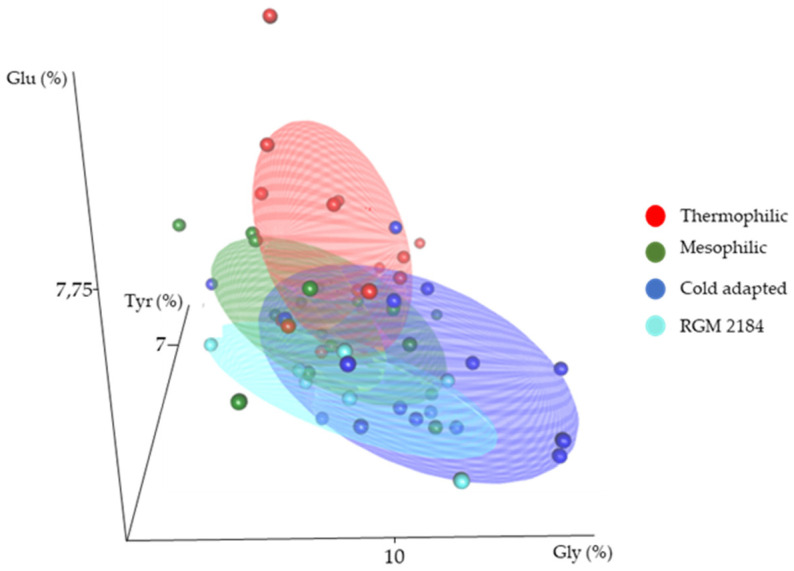
Three-dimensional scatterplot of cold-adapted (blue), mesophilic (green), and thermophilic (red) enzymes according to their amino acid composition, and identified enzymes secreted by *B. pseudobassiana* RGM 2184 (light blue). The axes correspond to the percentage of tyrosine (Tyr (%)), glutamic acid (Glu (%)), and glycine (Gly (%)) in the primary structure of enzymes.

An analysis of the amino acid composition between cold-adapted, mesophilic, and thermophilic enzymes indicated than cold-adapted protein enzymes had significantly more glycine [43] and less glutamic acid [44] and tyrosine than mesophilic and thermophilic enzymes (Figure 8). A comparative analysis of these features grouped the RGM 2184 exoenzyme close to the cold-adapted proteins, suggesting that this protein can adapt to low temperature (Figure 8).

Finally, future analyses of the purification, characterization, and expression of these exoenzymes will allow us to understand their role in the biocontrol of *B. pseudobassiana* RGM 2184 and to find future biotechnological uses for them.

## 4. Conclusions

The *B. pseudobassiana* RGM 2184 exolipases and chitinases identified in this study have been poorly characterized in entomopathogenic fungi. These exoenzymes can degrade the cocoons and pupae of *L. botrana* in the autumn and winter seasons. Additionally, they offer a competitive advantage over other control strategies, such as chemical insecticides, that cannot penetrate the cocoon.

## Data Availability

This whole-genome shotgun project has been deposited at DDBJ/ENA/GenBank under accession no. JAKJXD000000000.1. The nucleotide sequences of detected enzymes have been deposited in Genbank under accession n OP328424, OP328429, OP328425, OP328426, OP328430, OP328427, and OP328428, for P1, P2, Lip1, Lip2, Chi1, Chi2, and Chi3 exoenzymes, respectively.

## Supplementary Materials

The following supporting information can be downloaded at https://www.mdpi.com/article/10.3390/jof8101083/s1: Figure S1. Representative images of EPF *Beauveria pseudobassiana* colonization on pupae and cocoon of *Lobesia botrana.* Figure S2. Screening of enzymatic activity of protein extracts from supernatants of strain RGM 2184 culture grown in various media. Figure S3. Phylogenetic tree of exoproteases, exolipases, and exochitinases described and/or predicted in entomopathogenic fungi. Table S1. GenBank accession number of entomopathogenic fungus genome used in this study. Table S2. Analysis of amino acid composition of thermophilic, mesophilic, and cold-adapted proteins and exoenzyme of strain RGM 2184. References [45,46,47,48,49,50,51,52,53,54,55,56,57,58,59,60,61,62,63,64,65,66,67,68,69,70,71,72,73,74,75,76,77,78,79,80,81,82,83,84,85,86,87] are cited in the Supplementary Materials.
