# Supplementary material for: Identification of Exoenzymes Secreted by Entomopathogenic Fungus Beauveria pseudobassiana RGM 2184 and Their Effect on the Degradation of Cocoons and Pupae of Quarantine Pest Lobesia botrana
Source: J Fungi (Basel). 2022 Oct 14;8(10):1083. doi: 10.3390/jof8101083 (PMC9605004; doi:10.3390/jof8101083)
Supplement: Supplementary file 1 [file jof-08-01083-s001.zip › Figure S1.pdf]

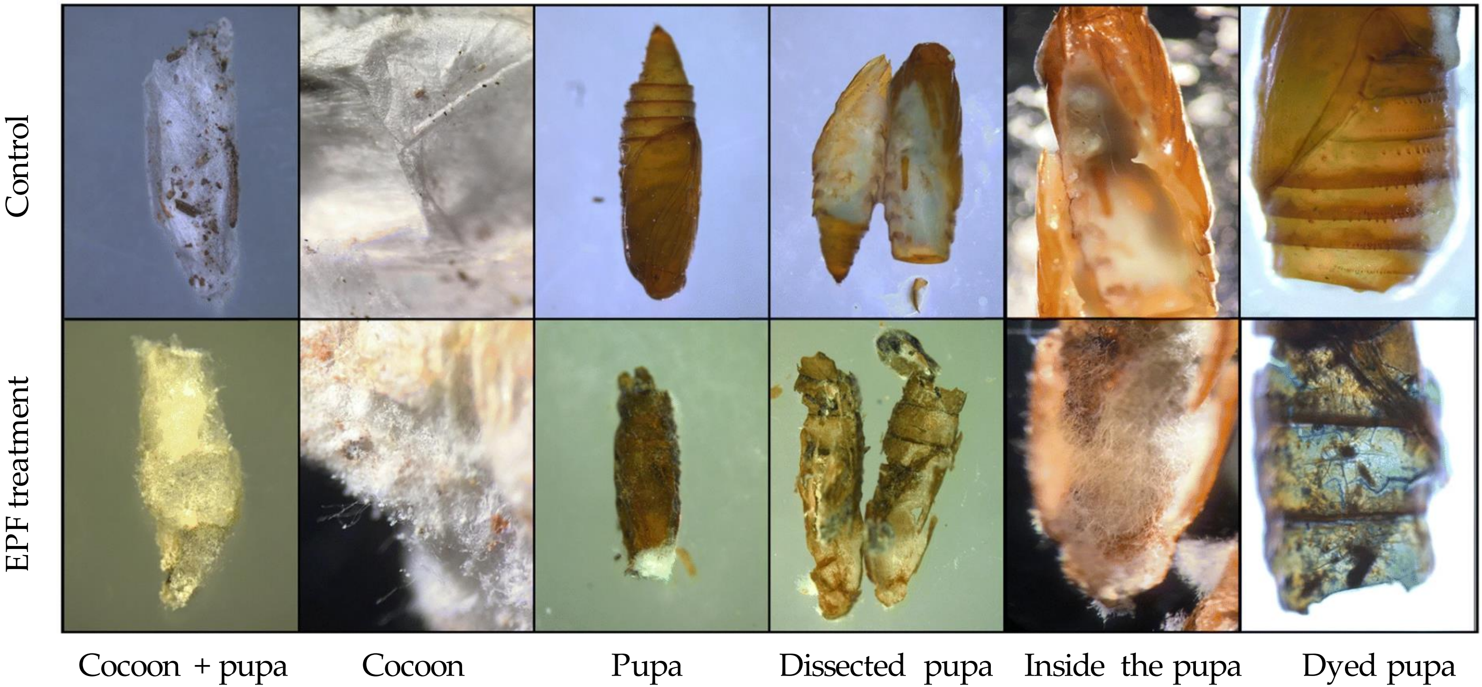

Figure S1. Representative images of EPF *Beauveria pseudobassiana* colonization on pupae and cocoon of *Lobesia botrana*. In the first row, images of a pupa subjected to the control treatment are shown. The lower row shows images of a pupa infected through *B. pseudobassiana* formulation treatment in the urban sector. The hyphae that penetrated the cuticle of the pupa are stained with lactophenol blue. Figure obtained from Altimira et al. [87].
