# Supplementary material for: Identification of Exoenzymes Secreted by Entomopathogenic Fungus Beauveria pseudobassiana RGM 2184 and Their Effect on the Degradation of Cocoons and Pupae of Quarantine Pest Lobesia botrana
Source: J Fungi (Basel). 2022 Oct 14;8(10):1083. doi: 10.3390/jof8101083 (PMC9605004; doi:10.3390/jof8101083)
Supplement: Supplementary file 1 [file jof-08-01083-s001.zip › Figure S2.pdf]

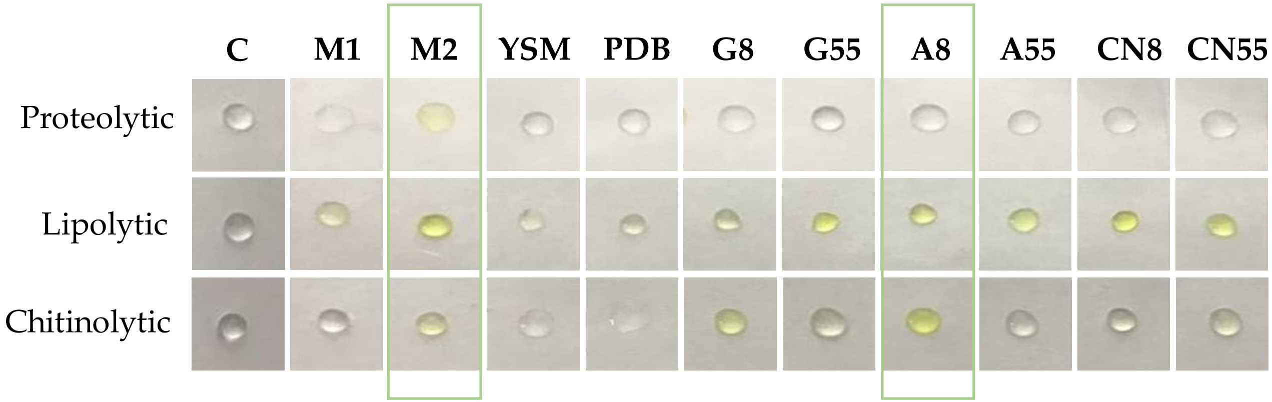

Figure S2. Screening of enzyme activity in protein extracts obtained from the supernatant of strain RGM 2184 in different culture media. Rows indicate colorimetric enzymatic activity assay. Columns indicate protein from supernatant extract of RGM 2184 in the ten cultures media. Green rectangles indicate protein extract selected.
