# Supplementary material for: Identification of Exoenzymes Secreted by Entomopathogenic Fungus Beauveria pseudobassiana RGM 2184 and Their Effect on the Degradation of Cocoons and Pupae of Quarantine Pest Lobesia botrana
Source: J Fungi (Basel). 2022 Oct 14;8(10):1083. doi: 10.3390/jof8101083 (PMC9605004; doi:10.3390/jof8101083)
Supplement: Supplementary file 1 [file jof-08-01083-s001.zip › Figure S3.pdf]

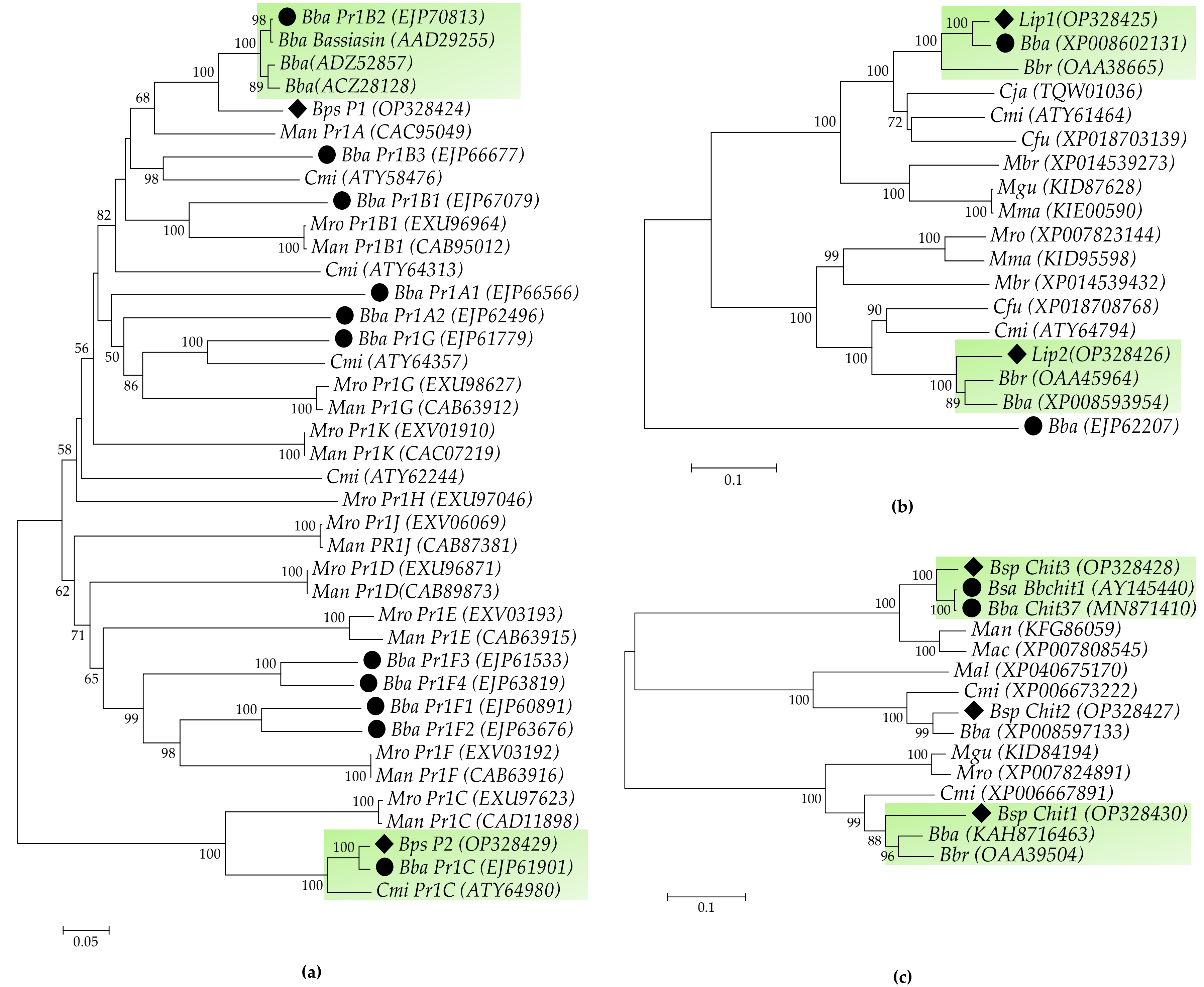

Figure S3. Phylogenetic tree of described and/or predicted exoproteases, exolipases, and exochitinases in entomopathogenic fungi. Phylogenetic trees of (a) exoproteases, (b) exolipases and (c) exochitinases were constructed using the neighbor-joining method in MEGA7 software. The trees are drawn to scale, with branch lengths measured by the number of substitutions per site. Enzymes identified in this study are indicated by diamonds. Exoenzymes described in other studies are indicated with a circle [32, 41, 42, 45-47]. At the end of the branch is the abbreviated species of the EPF strain followed by its NCBI accession code in parentheses. EPF species are abbreviated as follows: *Bba, Beauveria bassiana. Bbr, Beauveria brongniartii. Bps, Beauveria pseudobassiana. Cfu, Cordyceps fumosorosea. Cja, Cordyceps javanica. Cmi, Cordyceps militaris. Mal, Metarhizium album. Mgu, Metarhizium guizhouense. Mma, Metarhizium majus. Man, Metarhizium anisopliae. Mro, Metarhizium robertsii.*
