# Supplementary material for: Identification of Exoenzymes Secreted by Entomopathogenic Fungus Beauveria pseudobassiana RGM 2184 and Their Effect on the Degradation of Cocoons and Pupae of Quarantine Pest Lobesia botrana
Source: J Fungi (Basel). 2022 Oct 14;8(10):1083. doi: 10.3390/jof8101083 (PMC9605004; doi:10.3390/jof8101083)
Supplement: Supplementary file 1 [file jof-08-01083-s001.zip › Table S1.pdf]

Table S1. List of entomopathogenic fungal genomes used for LS-BSR analysis.

Strain	GenBank assembly accession
Purpureocillium lilacinum PLFJ-1	GCA_001653265.1
Cordyceps javanica IJ1G	GCA_006981985.1
Sporothrix insectorum RCEF264	GCA_001636815.1
Moelleriella libera RCEF2490	GCA_001636675.1
Metarhizium robertsii ARSEF2575	GCA_000591435.1
Lecanicillium psalliotae HWLR35	GCA_002796755.1
Akanthomyces lecanii UM487	GCA_001653215.1
Metarhizium acridum CQMa102	GCA_000187405.1
Cordyceps fumosorosea ARSEF2679	GCA_001636725.1
Metarhizium robertsii ARSEF23	GCA_000187425.2
Beauveria bassiana ARSEF1520	GCA_001684195.1
Beauveria brongniartii RCEF3172	GCA_001636735.1
Tolypocladium paradoxum NRBC100945	GCA_002916505.1
Aspergillus flavus NRRL3357	GCA_014117465.1
Beauveria bassiana ARSEF2860	GCA_000280675.1
Aspergillus flavus NRRL118543	GCA_014117465.1
Metarhizium anisopliae ARSEF549	GCA_002456175.2
Metarhizium rileyi Cep018-CH2	GCA_007866325.1
Beauveria pseudobassiana KACC47484	GCA_003267905.1
Beauveria pseudobassiana RGM2184	GCA_022985055.1
Metarhizium brunneum ARSEF3297	GCA_000814965.1
Metarhizium anisopliae BRIP53293	GCA_000426965.1
Metarhizium album ARSEF1941	GCA_000804445.1
Cordyceps javanica IJ2G	GCA_006981975.1
Ophiocordyceps polyrhachis-furcata BCC54312	GCA_001633055.2
Akanthomyces lecanii RCEF1005	GCA_001636795.1
Cordyceps militaris CM01	GCA_000225605.1
