# Supplementary material for: Identification of Exoenzymes Secreted by Entomopathogenic Fungus Beauveria pseudobassiana RGM 2184 and Their Effect on the Degradation of Cocoons and Pupae of Quarantine Pest Lobesia botrana
Source: J Fungi (Basel). 2022 Oct 14;8(10):1083. doi: 10.3390/jof8101083 (PMC9605004; doi:10.3390/jof8101083)
Supplement: Supplementary file 1 [file jof-08-01083-s001.zip › Table S2.pdf]

Table S2. List of enzymes used for aminoacid composition analysis.

	Activity	Kingdom	Organism	Accession number	Reference
**Cold-adapted**	Serine protease	Fungi	*Glaciozyma antarctica* PI12	AYC49704.1^{G}	[48]
			Linnemannia hyalina SCG-10	KAG9065897.1^{G}	[49]
			Glaciozyma antarctica PI12	CAQ76821.1^{G}	[50]
		Bacteria	*Vibrio* sp. PA-44	AAO16017.1^{G}	[51]
			Shewanella piezotolerans WP3 / JCM 13877	WP_020914201^{R}	[52]
			Colwellia psychrerythraea 34H / ATCC BAA-681	WP_011043668.1^{R}	[53]
	Lipase	Fungi	*Onygenales* sp. P7	ADY17230.1^{G}	[54]
			Emericella nidulans NFCCI 3643	EAA64964.1^{G}	[55]
		Bacteria	*Pseudomonas fluorescens* B68	AAU10321.1^{G}	[56]
			Bacillus pumilus 1_16A	WP_065098612.1^{R}	[57]
	Chitinase	Fungi	*Glaciozyma antarctica* PI12	AEH16745.1^{G}	[58]
			Linnemannia hyalina SCG-10	KAG9072959.1^{G}	[59]
		Bacteria	*Vibrio* sp. Fi:7	AAG12973.1^{G}	[60]
			Pseudoalteromonas sp. DL-6	QBJ64793.1^{G}	[61]
**Mesophilic**	Serine protease	Fungi	*Parengyodontium album*	1IC6^{P}	[62]
			Metarhizium acridum CQMa 102	XP_007814452.1^{R}	[63]
			Metarhizium robertsii ARSEF 23	XP_011411569.1^{R}	[63]
		Bacteria	*Staphylococcus epidermidis* Tu 3298	CAA44257.1^{G}	[64]
			Enterococcus faecalis MMH594	AAK67268.1^{G}	[65]
			Clostridium sp. BNL1100	AEY65929.1^{G}	[66]
	Lipase	Fungi	*Tuber melanosporum* Mel28	5MII^{P}	[67]
			Rhizopus delemar RA 99-880	EIE86673.1^{G}	[68]
		Bacteria	*Bacillus subtilis* 168	NP_388152.1^{R}	[69]
			Proteus mirabilis PM185	OZS69007.1^{G}	[70]
			Bacillus pumilus F3	ABK80759.1^{G}	[71]
	Chitinase	Fungi	*Trichoderma reesei* PC-3-7	OTA05027.1^{G}	[72]
			Trichoderma reesei PC-3-8	BAD44715.1^{G}	[72]
			Aspergillus niger An76	GAQ37864.1^{G}	[73]
			Trichoderma viride LTR-2	ABR27743.1^{G}	[74]
		Bacteria	*Bacillus circulans* WL-12	1ITX^{P}	[75]
			Paenibacillus pasadenensis CS0611	ASO66300.1^{G}	[76]
**Thermophilic**	Serine protease	Fungi	*Thermus thermophilus* JCM10941	BDA37360.1^{G}	[77]
			Thermoascus aurantiacus var. Levisporus	ABY71274.1^{G}	[78]
		Bacteria	*Pyrolobus fumarii* 1A	AEM38280.1^{G}	[79]
			Thermococcus kodakaraensis	WP_011250640.1^{R}	[80]
	Lipase	Fungi	*Thermomyces lanuginosus* T03	ABV69591.1^{G}	[81]
			Thermothelomyces thermophilus ATCC 42464	XP_003664847.1^{R}	[82]
			Thermothelomyces thermophilus ATCC 42464	XP_003659962.1^{R}	[82]
		Bacteria	*Geobacillus* sp.	WP_014195917.1^{R}	[83]
			Geobacillus sp.	WP_033008930.1^{R}	[83]
	Chitinase	Fungi	*Thermochaetoides thermophila* DSM 1495	XP_006692378.1^{R}	[84]
			Thermoascus aurantiacus var. Aurantiacus	ADT82646.1^{G}	[85]
			Thermochaetoides thermophila DSM 1495	XP_006692378.1^{R}	[84]
			Thermoascus aurantiacus var. Levisporus	ABS00927.1^{G}	[85]
		Bacteria	*Acetivibrio thermocellus* ATCC 27405	CAA93150.1^{G}	[86]

^{G} Genbank accession number; ^{R} NCBI Reference Sequence accession number; ^{P} Protein Data Bank structure ID.
