# Supplementary material for: Identification of Exoenzymes Secreted by Entomopathogenic Fungus Beauveria pseudobassiana RGM 2184 and Their Effect on the Degradation of Cocoons and Pupae of Quarantine Pest Lobesia botrana
Source: J Fungi (Basel). 2022 Oct 14;8(10):1083. doi: 10.3390/jof8101083 (PMC9605004; doi:10.3390/jof8101083)
Supplement: Supplementary file 1 [file jof-08-01083-s001.zip › Table S3.pdf]

Table S3. Average percentage of each amino acid for cold-adapted, mesophilic and thermophilic enzymes. Last three columns indicate the q value of the two-stage linear step-up procedure of Benjamini, Krieger, and Yekutieli.

aa	Cold adapted (%)			Mesophilic (%)			Thermophilic (%)			Cold-adapted vs Mesophilic	Cold-adapted vs Thermophilic	Mesophilic vs Thermophilic
	$\bar{x}$		σ	$\bar{x}$		σ	$\bar{x}$		σ	*q* value	*q* value	*q* value
**Ala**	10.28	±	2.7	9.01	±	2.5	9.72	±	2.1	0.54024	0.405918	0.866327
**Cys**	1.12	±	0.6	1.05	±	1.0	1.01	±	0.6	0.89454	0.663848	0.902428
**Asp**	6.04	±	1.6	6.28	±	1.3	5.97	±	1.3	0.856991	0.75692	0.741512
**Glu**	2.98	±	1.4	3.33	±	1.5	5.29	±	2.1	0.856991	**0.005671**	0.033267
**Phe**	3.39	±	0.9	3.45	±	0.8	3.67	±	0.9	0.89454	0.438019	0.701969
**Gly**	10.93	±	1.9	8.84	±	1.8	8.85	±	1.1	**0.007839**	**0.003756**	0.902428
**His**	1.67	±	0.8	1.88	±	0.9	2.04	±	1.0	0.856991	0.431052	0.902428
**Ile**	5.01	±	1.1	5.55	±	1.5	4.50	±	1.6	0.692372	0.681556	0.522053
**Lys**	3.75	±	1.7	5.79	±	2.9	4.14	±	1.7	0.133615	0.438019	0.500879
**Leu**	7.55	±	2.3	7.81	±	1.8	8.59	±	1.7	0.856991	0.319242	0.547268
**Met**	1.81	±	0.7	1.79	±	0.7	1.76	±	0.9	0.932639	0.731372	0.902428
**Asn**	5.49	±	1.5	6.04	±	2.1	4.89	±	1.8	0.856991	0.552061	0.522053
**Pro**	4.56	±	1.9	4.84	±	2.3	6.17	±	1.5	0.856991	0.070423	0.522053
**Gln**	3.28	±	1.1	3.10	±	1.1	2.94	±	0.9	0.856991	0.317612	0.688192
**Arg**	3.24	±	1.3	3.47	±	1.9	4.34	±	1.7	0.856991	0.149236	0.522053
**Ser**	8.69	±	2.2	8.67	±	2.2	6.04	±	1.4	0.934224	**0.003756**	**0.008768**
**Thr**	7.50	±	1.8	6.26	±	2.4	5.96	±	1.5	0.395353	0.052582	0.902428
**Val**	7.72	±	1.3	6.58	±	1.6	7.10	±	1.4	0.138528	0.232439	0.741512
**Trp**	1.42	±	0.9	1.63	±	1.0	2.02	±	1.0	0.856991	0.13347	0.522053
**Tyr**	3.57	±	0.7	4.64	±	1.2	5.01	±	1.1	0.039502	**0.001852**	0.522053

$\bar{x}$: mean; σ: standard deviation. **In bold** q value < 0.01.
